# Eosinophilic Cholecystitis as an Atypical Etiology of Biliary Colic: A Case Report and Review of the Literature

**DOI:** 10.7759/cureus.35831

**Published:** 2023-03-06

**Authors:** Mauricio Gutierrez-Alvarez, Santiago Vallarta, Rodrigo Cruz, Victor Visag-Castillo

**Affiliations:** 1 General Surgery, Medica Sur, Mexico City, MEX; 2 Department of Transplants, Instituto Nacional de Ciencias Médicas y Nutricion Salvador Zubirá, Mexico City, MEX; 3 Department of Transplants, Hospital General de México, Mexico City, MEX

**Keywords:** case report, abdominal pain, cholecystectomy, eosinophilic cholecystitis, cholecystitis

## Abstract

Eosinophilic cholecystitis (EC) is an uncommon cause of acute cholecystitis; the clinical presentation is indistinguishable from other types of cholecystitis, and the diagnosis is made by histopathology study. We present the case of a 73-year-old male patient with right hypochondrial abdominal pain suggestive of symptomatic cholelithiasis. There were no significant findings at the blood workup or physical examination; he underwent a cholecystectomy and was later diagnosed with eosinophilic cholecystitis by histopathology. EC may be associated with some other systemic conditions, such as eosinophilic granulomatosis, eosinophilic ascites, or parasitosis, which will require specific management.

## Introduction

Acute cholecystitis has a wide range of etiologies; among them, lithiasis accounts for 90% to 95% of cases [1,2]. Other types include acalculous cholecystitis, the less common emphysematous cholecystitis [3] and the even rarer eosinophilic cholecystitis (EC), which was first described in the French literature [4] by Albot in 1949 [5,6].

The literature has reported a prevalence of EC ranging from 0.25% to 6.4% [4]. More recently, Gutierrez-Moreno et al. reported a prevalence of 0.16% [6] in the Mexican population. Hypersensitivity or allergy to drugs such as penicillins, immunosuppression, and parasitosis, among others, have been proposed as associated risk factors [5,6]. On the other hand, some case series, such as the one reported by Khan et al. did not find any association with the previously described factors in their 22 patients diagnosed with EC [4], so the etiology still remains uncertain.

Clinically, EC is indistinguishable from other types of cholecystitis [4,5], the diagnosis is made with the pathology study in which the vesicular wall is infiltrated in >90% by eosinophils [5-7]. The course of the disease is determined by diseases associated with EC, such as eosinophilic granulomatosis, eosinophilic ascites, or parasitosis; because of this, it's important to check for any other disorders that may be related and treat them if necessary [5,6]. However, most of the time it takes a benign course.

## Case presentation

A 73-year-old male patient from Guerrero, Mexico, comes as an outpatient for medical evaluation at a private hospital in Mexico City. He has been presenting intermittent episodes of abdominal pain for seven days, predominantly in the epigastrium and right hypochondrium, colic type, intensity 10/10 on the pain scale, which is exacerbated with fatty meals and associated with nausea without vomiting; no previous management has been established. He has been diabetic for 30 years and is being managed with glimepiride and metformin. He has also smoked 20 cigarettes per day for 38 years. He also refers to being allergic to penicillin. Physical examination revealed good general condition. Abdomen without pain on palpation and no negative murphy sign. Rest of his examination including cardiovascular, respiratory and neurological showed no abnormalities.

**Figure 1:**
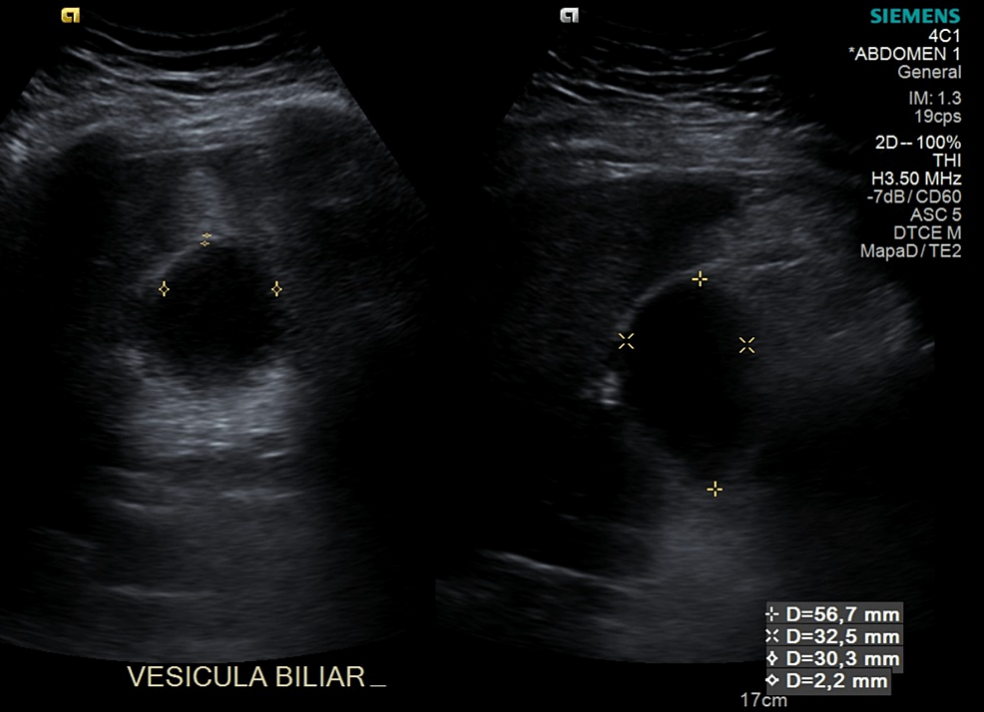
Gallbladder 56.7 x 32.5 x 30.3 mm with a wall of 2.2 mm, with no data of exacerbation at the time of the study.

**Figure 2:**
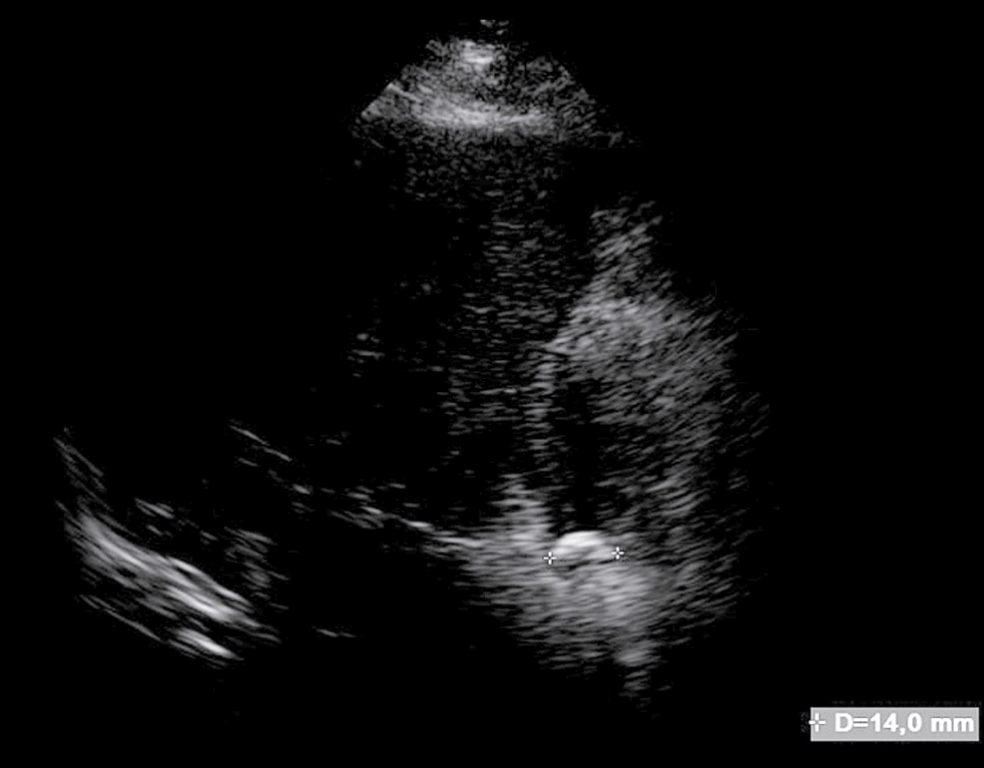
Image projecting posterior acoustic shadow in relation to 14 mm gallstone.

Liver and gallbladder ultrasound was requested as part of the diagnostic work-up where the findings were: gallbladder of 56.7 x 32.5 x 30.3 mm, thin wall of 2.2 mm, the content was an oval echogenic image which projects a posterior acoustic shadow with a diameter of 14 mm suggestive of one gallstone, choledochus of 3.1 mm (Figures 1, 2). His blood workup results were as follows: hemoglobin 12.4 g/dL, platelets 236,000 μl, leukocytes 10.800 μl, neutrophils 88% x 10³/L, lymphocytes 8%, eosinophils 0%, total bilirubin 0.62 mg/dL, alanine aminotransferase (ALT) 38 U/L, aspartate aminotransferase (AST) 45 U/L, alkaline phosphatase 137 U/L, gamma glutamyl transpeptidase 145 U/L, lactate dehydrogenase (LDH) 142 U/L. Based on these findings a diagnosis of symptomatic cholelithiasis was established with low risk of choledocholithiasis, so the patient was scheduled for a laparoscopic cholecystectomy.

**Figure 3:**
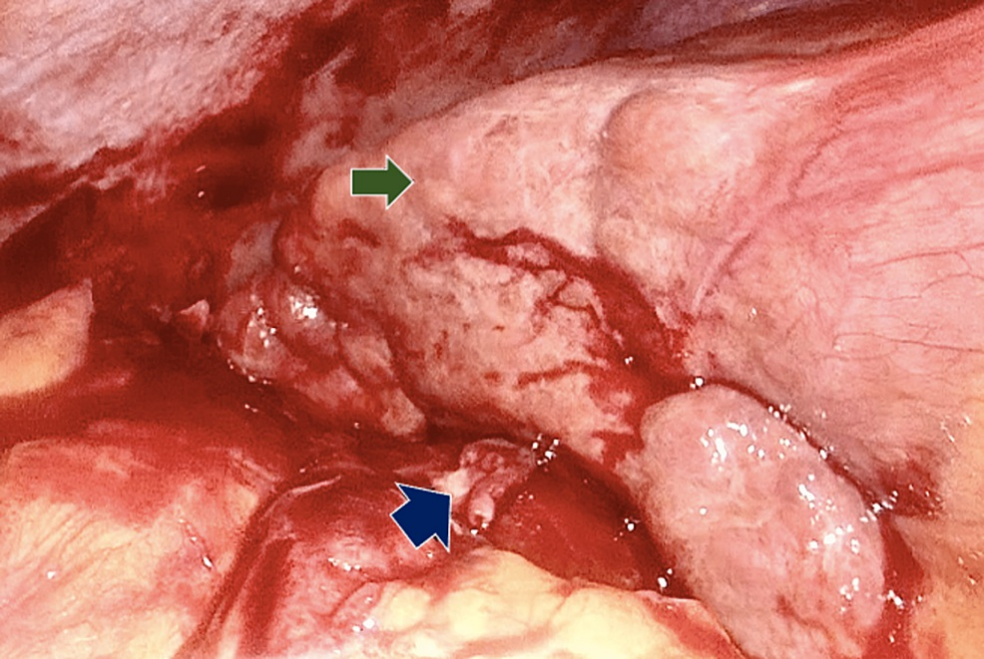
Green arrow liver with nodular appearance highly suggestive of liver cirrhosis. Blue arrow gallbladder with adhesions and omentum hiding it.

**Figure 4:**
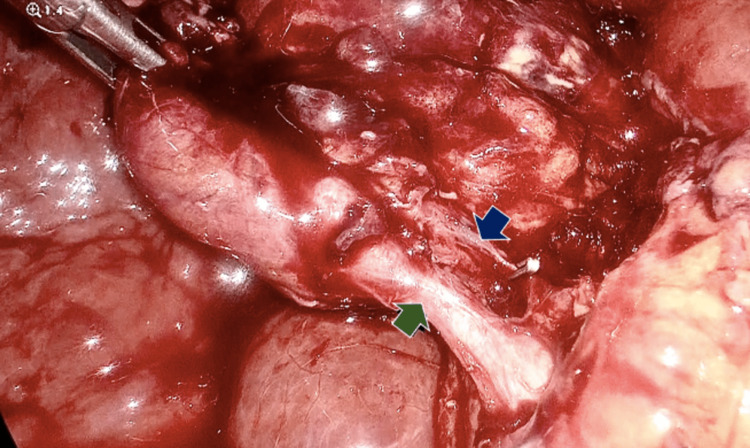
Blue arrow cystic artery with metal clip. Green arrow anterior cystic duct.

**Figure 5:**
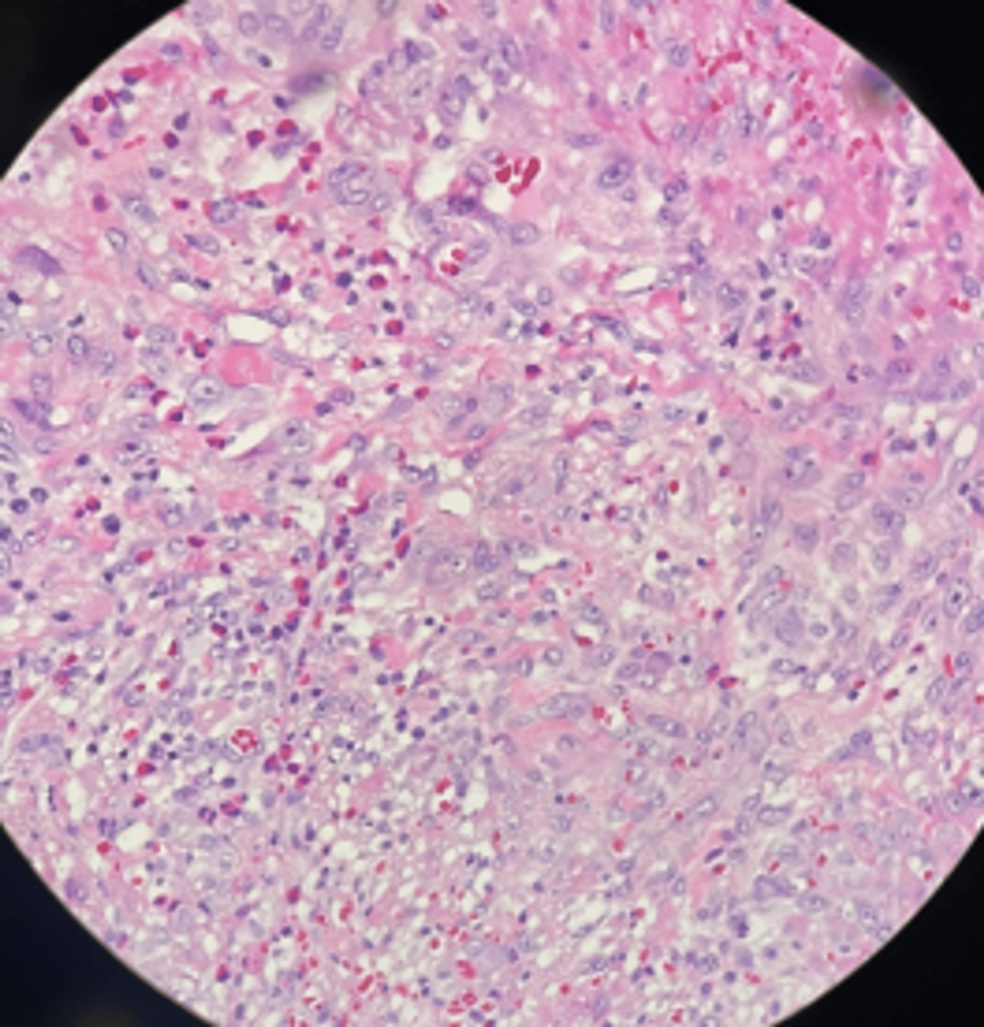
Gallbladder wall with inflammatory infiltrate showing numerous eosinophils. H&E 40x.

Surgical findings included firm adhesions of the omentum found burying the gallbladder, an inflamed gallbladder with necrotic areas, purulent liquid inside the gallbladder, a nodular liver, and a diminished size indicative of cirrhosis (Figures 3, 4). The specimen was sent to pathology, and days later the diagnosis of eosinophilic cholecystitis with extensive ulceration was reported. On the slides, >90% neutrophils were observed in a 40x field (Figure 5). A gallstone with a major axis of 1.2 cm is identified. After the procedure, the patient was discharged after 72 hours without any issues. Six months of patient follow-up revealed a suitable progression without problems and the absence of any additional diseases. The patient was directly asked for any other symptoms such as nasal allergies, sinus problems, rash, pain and numbness in your hands and feet, or any other gastrointestinal symptom. The patient did not accept to undergo any other additional diagnostic tool.

## Discussion

Cholecystitis is diagnosed in approximately 200,000 people per year in the US [1] and of these cases, the prevalence of EC in most of the literature is less than 1% [8] making it an uncommon condition. Reviewing case series in the literature, EC occurs in most cases around 37 years of age and with a higher prevalence in the female sex [4,6] unlike our case. A series described in the literature in pediatric patients similarly showed that the majority were female patients with a ratio of 1.5:1 [9].

The nature of this pathology remains uncertain, since although some authors propose the association of EC with allergies, including penicillin, as in the case of our patient [5,6], other authors haven't found any association [4], in the case of our patient, there was only a history of allergy to penicillin. In our case, maybe liver cirrhosis could be related, however there are no associations reported in the literature.

The most recent Tokyo guidelines suggest that pain in the right hypochondrium, Murphy's sign, temperature >38ºC, leukocytosis, elevated C-reactive protein (CRP), and findings in imaging studies have a sensitivity of 91.2% and specificity of 96.9% for cholecystitis [10]. More recent publications similarly suggest the use of a detailed clinical history, physical examination, laboratory studies, and imaging studies together to make the diagnosis of acute cholecystitis [1]. Clinically, however, EC resembles acute calculous cholecystitis [4,5], and the two are indistinguishable [6]. Therefore, physicians should be alert to any other symptoms that may suggest associated diseases, which should be questioned in a targeted manner when obtaining a definitive pathology result.

For EC, the definitive diagnosis is made through the histopathology report, where >90% of the gallbladder wall is infiltrated by eosinophils [5-7]. It can present with or without gallstones [4,5] as in our case, which presented a 14-mm gallstone. It should be remembered that peripheral eosinophilia is not present in most cases [5]. In cases where it is present, there should be a higher index of suspicion because there could be involvement of other organs and systems, as in the case reported by Ito et al where they describe the case of a patient with EC associated with eosinophilic granulomatosis who also showed increased eosinophils in the complete blood count [7].

EC is usually a benign condition; in those patients who only present inflammation of the gallbladder, it resolves with the surgical intervention "cholecystectomy" [5-7]. However, it will be necessary to look for some other associated condition intentionally to treat it. Cases of EC associated with eosinophilic granulomatosis and polyangiitis have been reported [7], which makes it necessary to provide systemic treatment to avoid complications derived from these associated pathologies. On the other hand, there are associations with eosinophilic inflammation of the gastrointestinal tract [11,12] and even airway inflammation [11], eosinophilic granulomatous hepatitis, and eosinophilic ascites [12]. Kaji et al. reported the case of a woman with EC and pericarditis; here, it is noteworthy that she presented high antibody titers against Ascaris lumbricoides and an adequate therapeutic response to management with albendazole alone [13]. One of our limitations is that since the patient presented an adequate post-surgical evolution, he did not accept to undergo any additional diagnostic tools to search for other diseases. However, the characteristics of cirrhosis in the liver could open lines of investigation to look for an association between both conditions.

## Conclusions

Eosinophilic cholecystitis is a rare diagnosis with a benign course that will require a cholecystectomy as part of the management. However, since the diagnosis can only be made postoperatively through the histopathological report, it is the task of physicians to intentionally look for other associated pathologies because EC has been associated with some other syndromes and diseases that will require specific management to avoid possible complications.
